# Combining *in silico* and *in vitro* experiments to characterize the role of fascicle twist in the Achilles tendon

**DOI:** 10.1038/s41598-018-31587-z

**Published:** 2018-09-14

**Authors:** Vickie B. Shim, Geoff G. Handsfield, Justin W. Fernandez, David G. Lloyd, Thor F. Besier

**Affiliations:** 1 Auckland Bioengineering Institute, University of Auckland, Auckland, New Zealand; 2 Innovations in Health Technology, Menzies Health Institute Queensland, Griffith University, Gold Coast Campus, Southport, Australia; 3 School of Allied Health Sciences, Griffith University, Gold Coast Campus, Southport, Australia; 4 Gold Coast Orthopaedics research and Education Alliance, Nathan, Australia; 5 School of Human Sciences, University of Western Australia, Perth, Australia; 6 Department of Engineering Science, University of Auckland, Auckland, New Zealand

## Abstract

The Achilles tendon (AT), the largest tendon in the human body has a unique structural feature, that is the fascicles in the AT display spiral twist. However, their functional and structural roles are still unclear. We used subject-specific computational models and tissue mechanical experiment to quantitatively characterize the role of fascicle twist in the Achilles tendon. Ten subject-specific finite element (FE) models of the Achilles tendon were developed from ultrasound images. Fascicle twist was implemented in these models using the material coordinate system available in our FE framework. Five different angles (0~60°) were implemented and material property optimization was performed for each of them (total 50 sets) using results from uniaxial stretch experiment. We showed that fascicle twist allows for even distribution of stress across the whole tendon, thus improving tissue strength. The predicted rupture load increased up to 40%. A number of connective tissues display similar fascicle twists in their structure. The resulting non-uniform strain distribution has been hypothesized as a primary factor in tissue degeneration and injuries. Therefore, our technique will be used to design biomechanically informed training and rehabilitation protocols for management of connective tissue injuries and degeneration.

## Introduction

The Achilles tendon is the largest tendon in the body and forms the mechanical link between the calf muscles and the calcaneus, playing a crucial role in the fundamental tasks of walking, running, and jumping. As a result, this tissue experiences high forces (up to 6~8 Body weights during running^1^) as well as substantial length changes^2^. The structure of this tendon is unique and well suited to perform high strain activities. It mainly consists of water (some 70% of the total weight) with the remainder being solid material in the extracellular matrix (ECM), of which 80% is type I collagen^3,4^. This matrix is organized into a hierarchical organization of collagen fibres in condensed parallel bundles.

An interesting feature of the Achilles tendon is that it is actuated by three muscles, that is medial gastrocnemius, lateral gastrocnemius and soleus. Fascicles from these muscles are fused distally to form a single tendon. Although they are tightly fused, the fascicles arising from these muscles form distinguishable bundles called sub tendons^5^. The three sub tendons (and the fascicles within the sub tendons) are twisted clockwise in the left limb and counterclockwise in the right, although the amount of rotation is variable across people^6^. The existence of fascicle twist was first described by Parsons in 1894^7^ and then later more comprehensively analyzed by White in 1943^8^ where he described a 90° twist of the tendon on its own longitudinal axis. Later van Gils *et al*.^9^ provided more comprehensive data on the nature of fascicle twist in the Achilles where they measured fascicle twist in 16 human cadaver specimens. One notable finding from this study was that the degree of tendon twist was smaller than 90° as previously reported by White and twist angles were specimen-specific, meaning that the twist angle formed a range between 11° to 65° with an average of 37°. Szaro *et al*.^10^ dissected 20 Achilles tendon specimens and showed that the sub-tendons associated with each of the triceps surae muscle bellies descend in a spiral fashion. More recently, Edama *et al*.^6^ provided results of morphological analysis from 60 cadavers (111 legs, Japanese males and females) and 53 cadavers (106 legs, Polish males), respectively, from which they classified the degree of fascicle twist, highlighting the twist variations between subjects.

However, what is missing in the current literature is an explanation of the functional role of the fascicle twist. Some authors have speculated that fascicle twist is nature’s way of increasing tendon strength while allowing the necessary elongation and elastic recoil^11^. Others have suggested that fascicle twist results in less fibre buckling, leading to reduced deformation of individual fibres under tension^9,12,13^. Yet, quantitative and systematic analysis of the role of fascicle twist in the Achilles tendon has not been conducted, and consequently there is currently no definitive answer to the question of why the human Achilles is twisted and how this twist influences tendon behavior. This knowledge is particularly important as recent studies have indicated that the nature of tendon deformation, specifically heterogeneous strain distributions, may contribute to the development of overuse injuries and tendinopathy^14,15^. The aim of this study was to determine the biomechanical role of fascicle twist in the Achilles tendon using experimental tissue mechanics and subject-specific computational finite element modelling. We hypothesised that the presence of fascicle twist allows for a more even distribution of stress across the whole tendon under the differential forces from the triceps surae muscles, thus improving tissue strength. Another important structural feature in the Achilles tendon is the sliding between sub-tendons^16^. We investigated this feature as well with a hypothesis that inter-sub-tendon sliding will improve force transmission within the tissue while fascicle twist redistributes stresses within the tissue.

We also performed sensitivity analysis to characterize how geometrical (tendon cross sectional area (CSA)) and structural features (fascicle twist and tendon stiffness) of the tendon affect the tissue strength. We hypothesize that CSA would have greater effects on tendon rupture load than either fascicle twist or tissue stiffness.

## Methods

### *In vitro* experiment

We used mechanical experimental data from a previous study by Wren, *et al*.^17^ who performed mechanical experiments with fresh-frozen human Achilles tendons from donors. The specimens were imaged with ultrasound and then underwent cyclic testing with a material testing machine (MTS, Eden Pairie, MN, USA). The tissues used in the experiment were from donors procured from the International Institute for Advancement of Medicine (Scranton, PA) and the Northern California Transplant Bank (San Rafael, CA). All experiments were performed in accordance with guidelines and regulations from these organizations. The current study used data from ten tendons (eight females and two males, average age of 68 years). The cross-sectional ultrasound images at 2, 4, and 6 cm proximal to the tendon’s calcaneal insertion were used to create subject-specific geometry of the tendon^18^. The force/displacement data from the cyclic testing was used to obtain subject-specific material properties as described below.

### Subject-specific computational models

A subject-specific finite element (FE) model was used to simulate fascicle twist in this current study. In our study, we followed the nomenclature proposed by Handsfield and colleagues^5^ and use the term fascicle to refer to the clearly defined units in the tissue that run along the length of the tendon and are visible to the eye with a diameter range of 50–400 μm. The FE modelling was conducted using CMISS (Continuum Mechanics, Image analysis, Signal processing and System identification), the computational framework developed as a part of the International Union of Physiological Society (IUPS) Physiome project^19,20^ that is freely available for academic use (http://physiomeproject.org/software/opencmiss). In our previous study, we generated ten subject-specific FE models using ultrasound images obtained as a part of the mechanical experiment described above^18^. The tendon FE model had a reference FE coordinate system (*x*_1_−*x*_2_−*x*_3_) and a material coordinate system with mutually-orthogonal curvilinear axes (*u*_1_−*u*_2_−*u*_3_), where *u*_1_ was aligned to the general fascicle direction of the tendon, which is proximal to distal directions. This resulted in FE models with straight fascicles.

### Continuum implementation of fascicle twist

We then implemented fascicle twist into our existing subject-specific straight fascicle FE models using a FE field fitting procedure^21^. Initially, fascicle twist was created by rotating and displacing the distal nodes in the straight fascicle model to reflect the fascicle twist as described in the literature^9,10^. The twisted model was overlaid with the original straight model, which allowed us to obtain corresponding coordinate axis vectors for both straight and twisted fascicles at any given material point in the tendon model. These were used to determine three Euler angles that aligned the material coordinate system of the straight fascicle model to the twisted fascicle model. A uniformly spaced dense data cloud was created in the straight fibre tendon model and the three Euler angles (non-zero angles that align directional vectors from the straight fibre to the twisted fibre) were determined for each data point and fitted as a FE nodal field, effectively generating fascicle twist as a field within the FE model. This field fitting procedure was repeated for four different twist angles: 15°, 30°, 45°, and 60°, for each subject specific tendon model. Since we had ten subject specific models, we generated 50 models in total, i.e. 10 models across five twist angles 0°, 15°, 30°, 45°, and 60°. The accuracy of fascicle twist implementation was evaluated against the data reported by Obst, *et al*.^22^. Using *in vivo* 3D ultrasound, they examined the degree of Achilles tendon transverse rotation from at rest to 70% maximum voluntary isometric ankle plantar flexion contraction. From 8 subjects they reported a mean rotation of 4.54 ± 2.49°. We subsequently, imposed a similar load in our models to test if these displayed similar transverse rotation. In this *in silico* experiment, we used the fascicle twist angle of 15°, which was the optimal angle found in our study. First, we converted the average ankle joint torque measured from Obst, *et al*.^22^ (i.e., 108.2 Nm) into a muscle force using the moment arm measurements reported by Fath, *et al*.^23^. This load was applied to our model and the transverse rotation was measured in the same manner as Obst and colleagues, which was to measure the angle of rotation with respect to the principal axis of the most distal cross section.

### Tendon material property characterization

Tendon material properties were estimated in a similar way to our previous work^18,24^, which described tendon as a transversely isotropic and hyperelastic material using the constitutive model developed by Gardiner and Weiss^25^. Since the deformation of the tissue is large, we used finite elasticity in our modelling framework. The material coefficients values were estimated using the cyclic experimental data from Wren, *et al*.^17^. This material model described tendon as a composite of ground substance matrix with embedded collagen fibres, i.e.

1 $$W={F}_{1}({I}_{1})+{F}_{2}(\lambda )$$

where *F*_1_ represents the contribution from ground substance as a function of *I*_1_, the first invariant of the right Cauchy-Green deformation tensor. *F*_2_ represents the contribution from the collagen fibres as a function of fibre stretch *λ*. The ground substance was described with the neo-Hookian model with one coefficient (C_1_) that was determined as the following:

2 $${F}_{1}=\frac{{C}_{1}}{2}({I}_{1}-3)$$

The strain energy of the collagen fibres, *F*_2_, was represented as a piecewise function that describes non-linear stress/strain behaviour as the following with four coefficients, C_3–6_.

3 $$\begin{array}{rcl}\lambda \frac{\partial {F}_{2}}{\partial \lambda } & = & 0,\,for\,\lambda \le 1,\\ \lambda \frac{\partial {F}_{2}}{\partial \lambda } & = & {C}_{3}[{e}^{{C}_{4}(\lambda -1)}-1],\,for\,1\le \lambda \le {\lambda }^{\ast }\\ \lambda \frac{\partial {F}_{2}}{\partial \lambda } & = & {C}_{5}\lambda +{C}_{6},\,for\,\lambda \ge {\lambda }^{\ast }\end{array}$$

where λ^*^ is the stretch value where the collagen fibres become uncrimped or straightened. Overall the material model has five material coefficients that had to be estimated, where *C*_1_ scales the ground substance stress, *C*_3_ scales the exponential stress for collagen fibres, *C*_4_ represents the rate of collagen fibre uncrimping, *C*_5_ is the modulus of the straightened collagen. The value for the modulus of the straightened (*C*_5_) collagen was obtained from the gradient of the experimental stress/strain curve, while three parameters (*C*_1_, *C*_3_ and *C*_4_) were estimated by performing material property optimization^26^. *C*_6_ was determined using the following relationship that ensures smooth transition between the 2^nd^ and 3^rd^ piecewise functions above

4 $${C}_{6}={C}_{3}(\exp ({C}_{4}({\lambda }^{\ast }-1))-1)-{C}_{5}{\lambda }^{\ast }$$

Optimization of *C*_1_, *C*_3_ and *C*_4_ was achieved by doing FE simulations of the experiments performed by Wren, *et al*.^17^. In this, the FE model boundary conditions replicated those in the experimental set up, where the distal nodes were fixed to represent the clamp that fixed the calcaneus, while proximal nodes were applied with the same amount of force used in the experiment. The tendons used in the experiment had 6 markers placed on the surface of the tendon. During cyclic stretch of the tendon, a CCD (Charge Coupled Device) camera recorded and tracked the movements of those markers, from which strain vs. time curve was generated. In our optimization, we also tracked the movements of those markers. Each subject-specific AT tendon model had markers placed on them in the same manner as the experimental tendons (6 markers placed in equidistance from distal to proximal end of the free tendon). The quality of fit was determined by calculating the RMS (Root Mean Square) error between the markers in the experimental tendon and those in the FE models. The *C*_1_, *C*_3_ and *C*_4_ values that produced the minimum RMS error were selected as the optimum material parameters for subsequent simulations. For more information on the material parameter optimization, see Shim, *et al*.^18^. We optimized parameters for all five twist angles considered (0°, 15°, 30°, 45°, 60°) for each tendon specimen, yielding a total of 50 sets of optimized parameters.

### Investigation of sub-tendon sliding

In order to characterize the role of sub-tendon sliding, we built a separate model of one of the subjects that incorporated the physical sub-tendon structures. Since the previous study by Handsfield *et al*.^16^ showed that the displacements of the medial gastrocnemius sub-tendon (MG sub-tendon) and lateral gastrocnemius sub-tendon (LG sub-tendon) are similar under passive muscle force, we built a sub-tendon sliding model consists of soleus sub-tendon and gastroc sub-tendon with 30° twist. Geometric fitting procedure was used to deform the model to capture the clockwise twist of fascicles within the tendon^27^. Four different cases were simulated to characterize the role of twist and sliding – (1) straight sub-tendon model with no sliding; (2) straight sub-tendon model with sliding; (3) twisted sub-tendon model with no sliding; (4) twisted sub-tendon model with sliding. The average material properties from material optimization were used. A uniaxial stretch was applied to all four models until failure and the changes in stress distribution pattern were analysed. The failure strain reported by Wren and colleagues^28^ was used to measure tissue strength. The resulting stress pattern from the sub-tendon sliding model was also compared with the results from the embedded fascicle twist model where the fascicle twists were implemented with fibre fitting procedure described above. It was to see if the embedded fascicle twist models can display the effects of sub-tendon sliding. Since both models have the same number of nodes and elements, we compared the stress values at nodal points to see if the results from these two cases are similar or not.

### *In silico* tendon rupture experiment

We investigated how fascicle twist altered tissue internal stresses by performing *in silico* experiment that predicted the tissue stress and rupture load for tendons with different fascicle twist angles. To account for the differential loading that the Achilles experiences during *in vivo* activities, we used force ratios consistent with physiological cross sectional areas (PCSA) ratios of triceps surae components reported in the literature^29^. These studies measured muscle volumes and PCSA of the triceps surae muscles with MR and showed that the ratio between soleus, medial gastrocnemius and lateral gastrocnemius to be approximately 6:2:1. This ratio is also consistent with previous models of differential loading in the Achilles tendon^16^. Finally, the rupture load was set to 100 MPa, as reported in other *in vitro* studies^28,30,31^.

In the *in silico* experiments the distal end of the model was fixed and force was applied to the proximal nodes, similar to material property optimization simulations. Forces were applied in steps of 100 N until the tendon model experienced von Mises stress greater than 100 MPa. We applied the total force such that two thirds of this was applied to fascicle nodes belonging to the soleus sub tendon, and the remaining force applied to the medial and lateral gastrocnemius sub tendons in a two-to-one ratio, to be consistent with the relative sizes of the triceps surae muscles^29^. The model was regarded to have ruptured when 15 or more consecutive Gauss points, corresponding to about 3 mm of length, had von Mises stress greater than 100 MPa. This *in silico* experiment was repeated for all 50 cases (10 specimen specific models across 5 different twist angles). Stress distribution pattern during the *in silico* experiment was also quantitatively analysed. We divided our model into medial and lateral compartments along the centre line of the model. This groups elements on the medial side and the lateral side separately into two separate regions. We compared how the stresses in these regions change depending on the twist angle. All computer simulations were done using the open-source based computational framework developed as a part of the International Union of Physiological Society (IUPS) Physiome project that is freely available for academic use (http://physiomeproject.org/software/opencmiss). Models developed will be deposited model repository for Physiome Project model repository in https://models.physiomeproject.org/welcome where users can run and view the result of the simulation using our software.

### Statistical and sensitivity analysis

Various other analyses and statistical tests were carried out on the data from the *in silico* experiments. The predicted rupture loads for different twist angles were compared using one-way ANOVA (Analysis of Variance) to test for significant differences (p < 0.05) between these twist cases. Sensitivity analyses were also performed to quantitatively characterize the influence of three major aspects of tissue mechanics, i.e. the (1) structural aspect, via changes in the fascicle twist angle; (2) geometric aspect, via changes in the cross sectional area (CSA); and (3) material property aspect, via changes in the collagen stiffness, i.e. *C*_5_. To this end, we used the means and standard deviations (σ) for these parameters reported in the literature as well as from the *in vitro* experiment. For stiffness (*C*_5_) and CSA, we used the *in vitro* experimental data from 43 cadavers^17,28^, while the twist angles data (n = 16) were from the study by van Gils, *et al*.^9^. The changes in CSA were simulated using our Free Form Deformation method^18^, which allowed the mid-section of the mesh to either shrink or expand by a given amount (in this case ±σ). This sensitivity analysis was performed on all ten subject-specific models used in this study. The original subject-specific model’s twist angle, CSA and stiffness values were varied by ±σ and the rupture load was measured as described above. The changes in the rupture load were recorded and compared to identify the factors that most influenced tendon strength.

## Results

**Figure 1:**
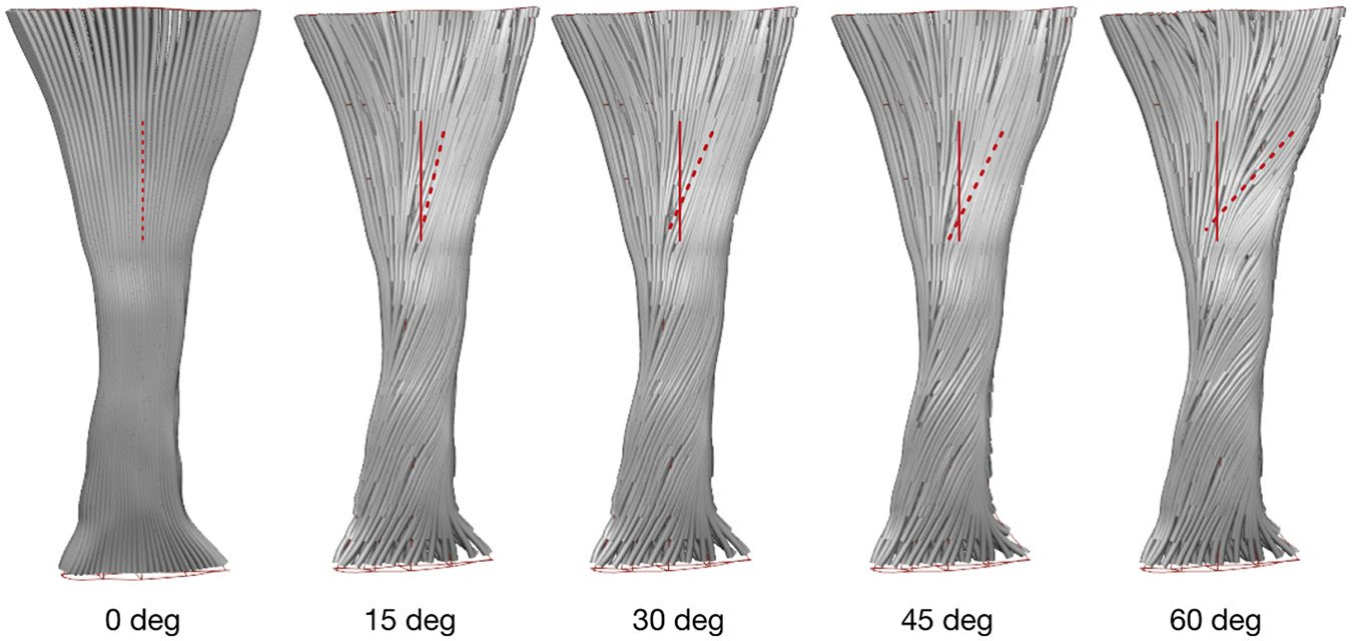
Fascicle twist implemented in FE models, illustrating an anterior view with medial on the left and lateral on the right. The red dotted lines indicate the degree of external rotation of the fascicles at different fascicle twist angles.

The fascicle twist angles implemented using the material coordinate system in our model are shown in Fig. 1, which shows the rotation of the fascicles in the tendon. The validity of our implementation was tested against the data from an *in vivo* experiment that measured transverse rotation of the Achilles tendon from at rest to 70% maximum voluntary isometric ankle plantar flexion contraction using 3D ultrasound imaging^22^. When subject to the similar loading condition to the study, our free Achilles tendon models displayed 6.89 ± 3.74° transverse rotation. This is comparable to the values reported *in vivo* measurements (4.54 ± 2.49°), indicating that the fascicle twists implemented in our models were representative of *in vivo* tendon structure and behaviour.

The optimized values for the material coefficients for the transversely isotropic material property of the tendon varied when twist angle changed across the specimens (Table 1). The average RMS error between the predicted and actual markers was 0.326 ± 0.096 mm (Table 2).

**Table 1:** Optimized material property coefficients for different twist angles.

Angle	C1(MPa)					C3(MPa)					C4				
Subjects	0°	15°	30°	45°	60°	0°	15°	30°	45°	60°	0°	15°	30°	45°	60°
1	38.07	38.18	46.00	75.39	78.00	11.57	12.92	13.00	3.41	9.10	30.65	30.67	24.00	1.12	9.00
2	22.74	23.24	34.16	29.34	89.54	13.23	21.95	5.97	6.03	9.75	42.03	33.82	64.28	69.02	5.92
3	43.13	30.15	27.34	29.82	29.81	2.21	6.28	1.64	2.91	3.08	97.37	70.23	124.02	99.77	99.79
4	49.92	46.37	55.02	46.39	46.33	14.05	11.71	9.20	11.86	12.20	42.19	53.45	56.27	53.53	53.68
5	164.18	146.57	148.07	146.52	143.10	31.73	36.18	37.29	34.98	37.37	2.95	2.68	2.40	2.81	2.91
6	46.52	42.62	39.07	41.84	21.76	15.29	3.34	5.76	3.79	14.37	26.80	62.70	50.43	60.17	39.57
7	40.20	14.28	40.06	14.65	31.62	4.98	4.06	5.82	4.82	1.79	83.34	127.14	80.12	117.72	155.45
8	35.73	12.79	14.54	11.27	6.92	6.22	3.09	2.92	3.33	4.25	73.12	100.00	99.83	100.00	100.00
9	25.38	24.68	21.97	23.40	21.61	7.90	1.68	2.76	2.50	3.77	14.59	83.82	70.14	74.02	64.76
10	98.95	142.59	142.78	140.35	143.10	27.06	35.88	35.62	33.70	35.49	26.15	3.55	3.53	4.11	3.65
Average	70.03	53.70	56.90	53.73	59.31	16.40	13.80	12.00	11.55	13.56	41.04	59.71	57.50	64.57	58.41
	54.19	52.74	39.72	52.11	52.84	15.17	14.06	11.04	13.23	13.67	36.20	41.81	36.78	40.37	52.57

C_1_ refers to the coefficient for ground substance, C_3_ is the scaling factor for exponential stress in collagen fibres, C_4_ is the rate of collagen fibre loading.

**Table 2:** RMS error between predicted and actual marker positions for all 10 subjects at five different twist angles.

Angle	0°	15°	30°	45°	60°
Subjects					
1	0.268	0.276	0.319	0.316	0.280
2	0.234	0.265	0.289	0.265	0.341
3	0.283	0.301	0.303	0.305	0.274
4	0.269	0.275	0.278	0.277	0.259
5	0.405	0.402	0.404	0.404	0.399
6	0.317	0.321	0.326	0.323	0.284
7	0.356	0.361	0.371	0.360	0.371
8	0.556	0.566	0.569	0.567	0.548
9	0.256	0.258	0.262	0.259	0.250
10	0.314	0.312	0.312	0.312	0.313
Average	0.326	0.334	0.343	0.339	0.332
SD	0.096	0.093	0.090	0.091	0.090

**Figure 2:**
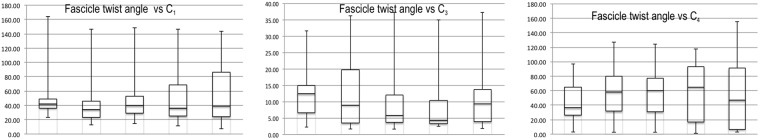
Optimized material property coefficients for different twist angles shown in a box and whisker plot. Results for C_4_ shows that models with fibre twist has a statistically higher rate for collagen fibre uncrimping (p = 0.04).

Material optimization results showed no statistically significant differences between optimized parameters with respect to twist angles for C_1_ & C_3_ (p = 0.47 for C_1_ & 0.22 for C_3_ at 30° twist). As for C_4_ (the material coefficient for collagen fascicle uncrimping, however, there was a statistically significant trend for fascicle twist models to have higher values than the non-twist model (p = 0.04 for C_4_ at 30° twist) (Fig. 2).

**Figure 3:**
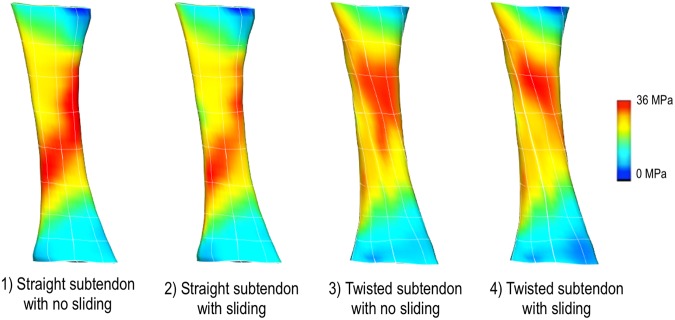
Comparison of stress distribution pattern for different sub-tendon configurations under 10% uniaxial stretch. When sub-tendon is twisted, the stress distribution pattern changes. When sliding is allowed, the distribution patterns did not change much. Rather the force transmission between sub-tendons is improved.

Sub-tendon sliding model revealed the roles that fascicle twist and sliding play in the tissue stress distribution. As can be seen from Fig. 3, fascicle twists redistributed stresses within the tissue while sliding improved force transmission between sub-tendons. When comparing failure strains between the models with and without sub-tendon sliding, the failure strain increased by 13% when sliding is incorporated into the model.

**Figure 4:**
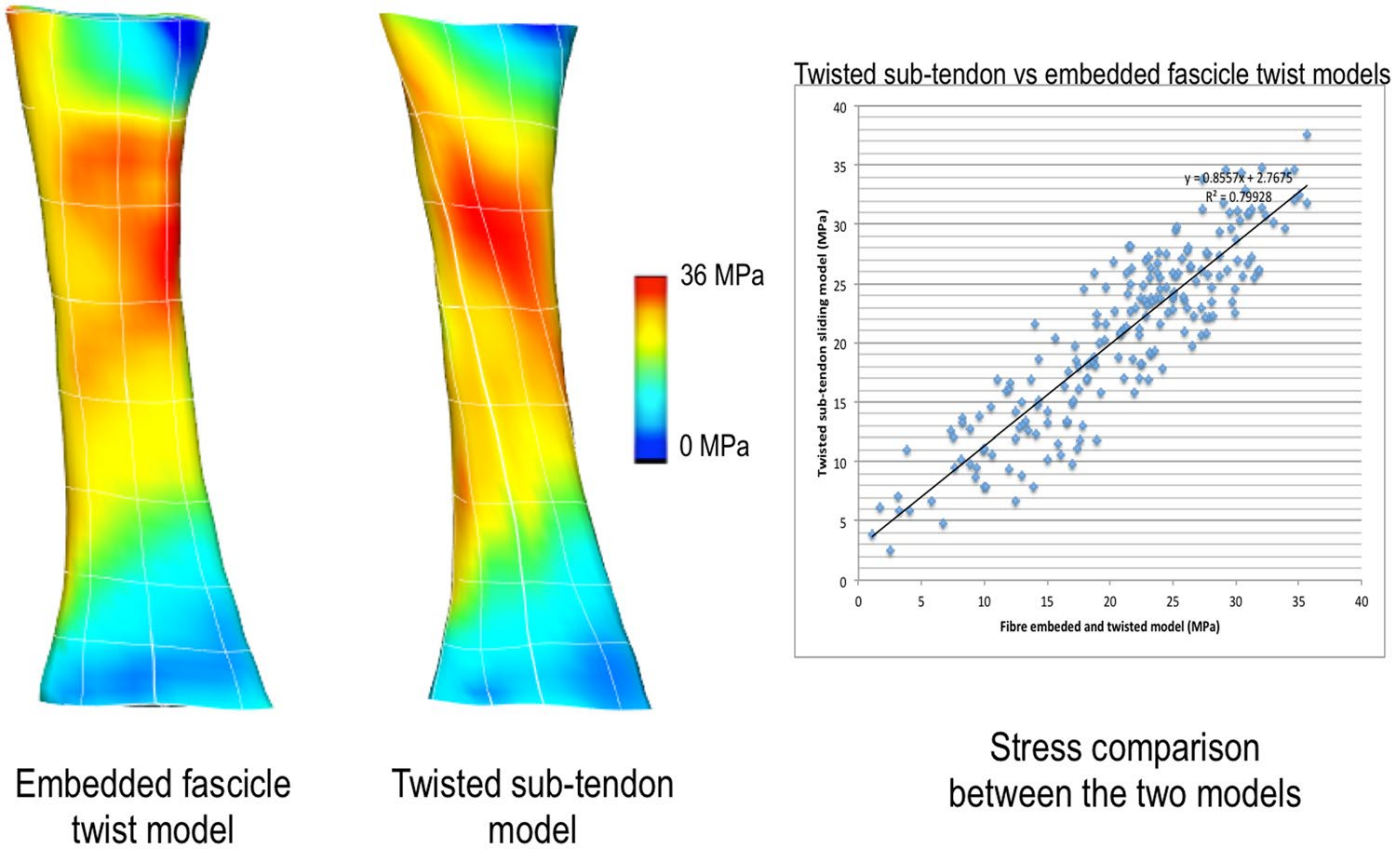
Comparison of stress distribution patterns between twisted sub-tendon model and embedded fascicle twist model. When stress values at nodal points were compared, the r^2^ was 0.8, indicating that these two models exhibit similar stress pattern.

When the results from the sub-tendon sliding model were compared with the model where the fascicles twist was embedded using the fibre fitting procedure, the results were similar (r^2^ = 0.8) showing that embedding fascicle twist to the model with fibre fitting procedure is capable of displaying the effects of sub-tendon sliding (Fig. 4).

**Figure 5:**
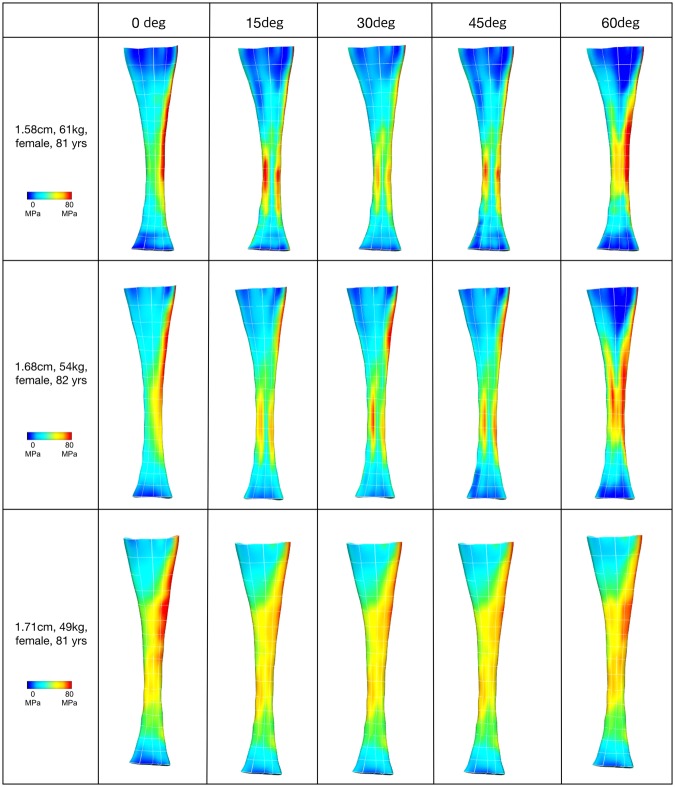
von Mises Stress distributions across five fascicle twist angles. Posterior view with medial on the right and lateral on the left. The presence of fibre twist changes the location of stress concentration from medial to both medial and lateral sides.

**Figure 6:**
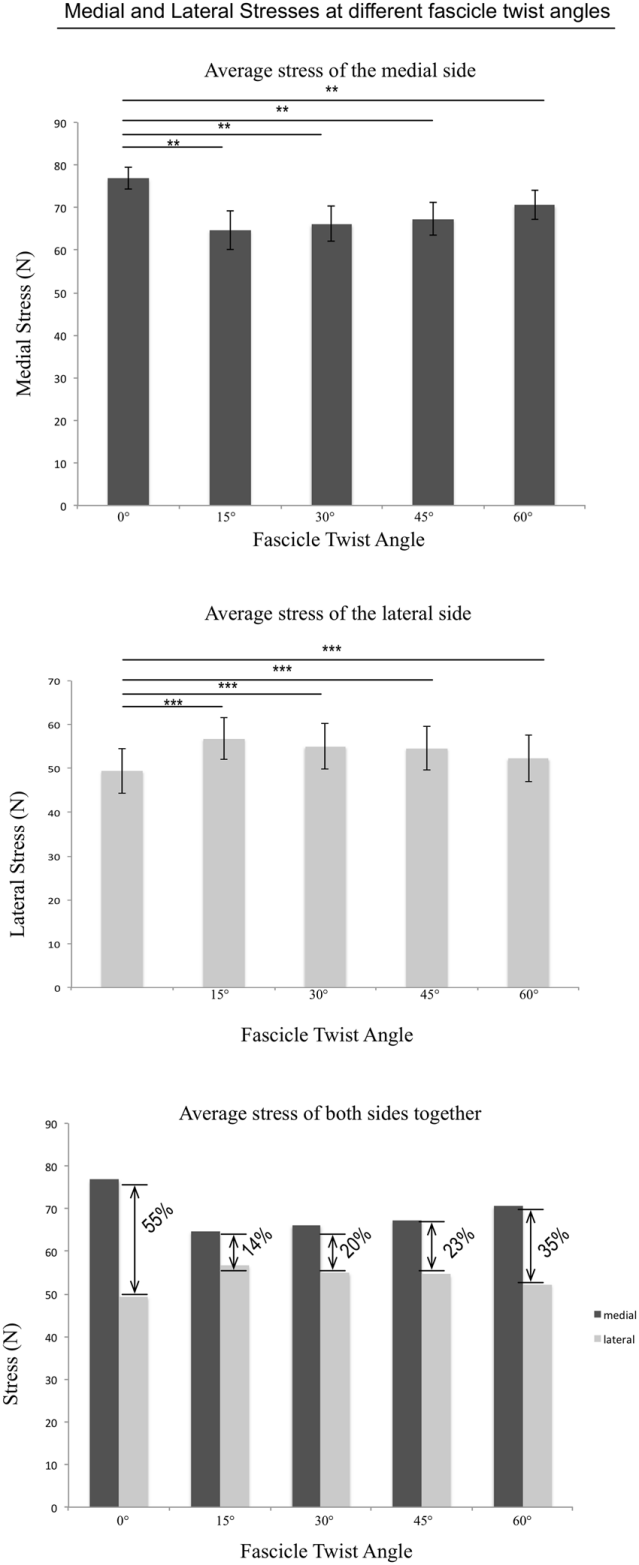
Comparison between average stresses from the medial and lateral sides of the tendon. When fascicle twists were present, both medial and lateral side stresses were significantly different compare to the no twist case. The percentage difference between the two reduced when twist was introduced and was the smallest when the twist angle was 15 degrees. n = 10 with *p < 0.05, **p < 0.01 and ***p < 0.001.

Stress distribution patterns varied with the different fascicle twist angles, as shown in three representative cases from the ten models used in the *in silico* experiment (Fig. 5). When no fascicle twist was present, stress was concentrated primarily on the medial side of the tendon. Increases in fascicle twist redistributed this stress concentration to both medial and lateral sides, essentially relieving stress concentration. The average stresses from medial and lateral sides of the tendon were computed separately and compared to quantify the effect of fascicle twists (Fig. 6). We computed the percentage difference between medial and lateral stresses at different fascicle twist angles and showed that when no twist is present, the average stress on the medial side is 55% larger than the lateral side. This difference was reduced when fascicle twist was present up to 14%.

**Figure 7:**
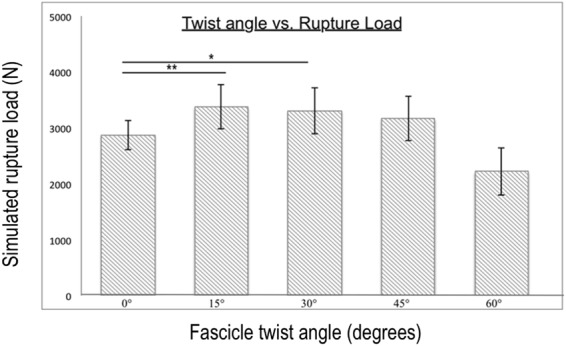
Compared to 0° fascicle twist, tendon strength is significantly improved by up to 30% when the angle is smaller than 30°. At 45° twist angles there is a nonsignificant larger rupture load compared to 0°, while 60° of twist there is a lower rupture load, suggesting a twist angle in the range of 15°–45° is optimal for tissue strength. n = 10 with *p < 0.05, **p < 0.01 and ***p < 0.001.

Simulated rupture loads from our *in silico* tendon rupture experiment revealed that fascicle twist angles of up to 30° improved Achilles tendon strength (Fig. 7). Compared to non-twist angle, there was 39.87% increase for 15° and 25.71% for 30° (Fig. 7). When considering the predicted rupture loads from all five cases, there was a range of twist angles between 10° and 30° that improves tendon strength.

**Figure 8:**
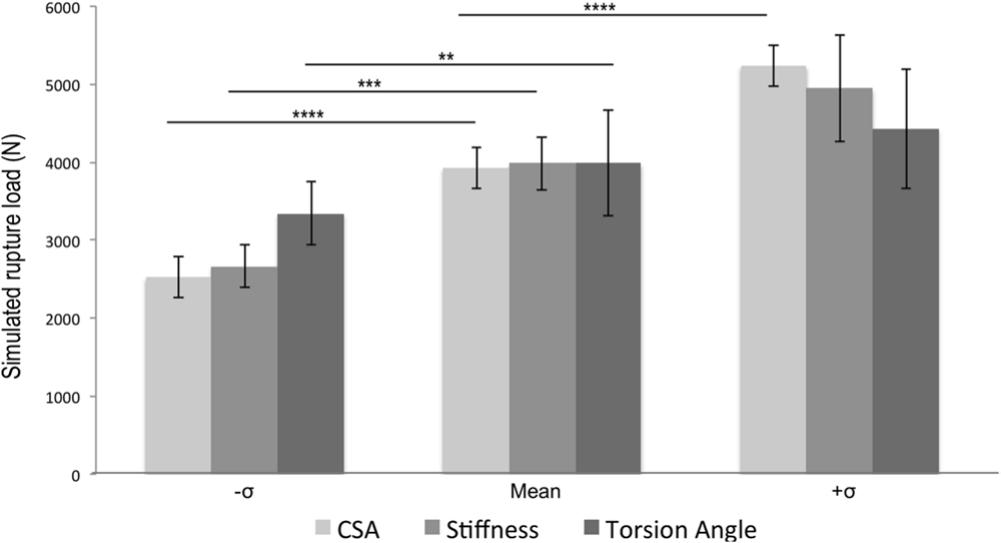
Sensitivity analysis reveals that tissue strength improves with increases in CSA, stiffness, and twist angle. Twist angle contributes less to tissue strength than either stiffness or CSA. n = 10 with *p < 0.05, **p < 0.01 and ***p < 0.001.

The results from sensitivity analyses show that changes in the parameters tested (CSA, stiffness, and twist angle) led to significant changes in rupture load. When decreased by one standard deviation from their mean values, the predicted rupture loads decreased in all three cases, but CSA decrease led to the greatest decrease in rupture load (mean 36% change, p < 0.0001), whereas changes to twist angle caused the least significant decrease (mean 15% change, p = 0.043) (Fig. 8). When increased by one standard deviation from their mean value, the predicted rupture load also increased in all three cases but only the changes in CSA led to statistical significance (33% increase in rupture load, p < 0.001).

## Discussion

In this study, we set out to understand the mechanical effects of fascicle twist in the Achilles tendon. We hypothesised that the presence of fascicle twist allows for even distribution of stress across the whole tendon in the presence of differential forces from the triceps surae muscles, thus improving tissue strength. Using subject specific FE analysis and data from *in vitro* tissue mechanical experiments, we showed that fascicle twist changed internal stress patterns, which in turn led to increased tissue strength, which was generally consistent with our hypothesis. The fact that our model estimated the *in vivo* transverse rotation of the Achilles tendon reported in Obst *et al*.^22^ gives us further confidence in our results.

The data available from the mechanical experiment allowed us to generate subject specific geometries for our FE models and we determined material properties using a parameter optimisation method. We found that fascicle twist plays a role in equalizing stresses within the tendon, specifically by distributing medial-side stress to the lateral free tendon. This may be particularly important because the motion of the foot from dorsiflexion eversion to plantarflexion inversion results in stress and strain concentrations on the medial tendon^32^. Consistent with this, in our models with no fascicle twist, we observed stress concentration on the medial tendon (no fascicle twist model in Fig. 5). Localized stress concentrations such as this will weaken the overall strength of the tissue and predispose the tissue to injury. Fascicle twist of up to 45° appears to redistribute medial stress concentrations to broader areas, reducing peak stress and relieving stress concentrations. Rupture loads increased up to 40%, compared to zero twist, when fascicle twist was present (Fig. 7) indicating that fascicle twist improved the effective strength of the tissue under *in-vivo* loading cases.

The twisted structure of the Achilles tendon has been investigated widely in the past^6,8–10,33^. However, the functional role of fascicle twist in the Achilles has not been elucidated despite this being an active area for discussion and scientific observation. Previous authors suggested that fascicle twist allows greater energy storage in the tendon, thus improving tissue strength^12,13,34^. In a recent study, Dean, *et al*.^35^ provided an interesting explanation of the role of tendon twist on muscle behaviour. Using the anterior jaw adductor of the spotted ratfish as a theoretical model, they elegantly showed that in broad muscles attached to rotating structures, fascicle twist in the tendon equalizes strain across the musculotendon complex (MTC), allowing higher force generation. If no twist were present, the fibre lengths within the muscle would vary greatly throughout the joint range of motion, leading to variable moment arms throughout the muscle and undermining the muscle’s contractile ability. Fascicle twist resolves this problem as the twisting minimizes variations in fibre strains. The human triceps surae is, in fact, a broad muscle that is attached to a rotating structure (calcaneus) through the Achilles tendon. Therefore, it is likely that the fascicle twist in the Achilles performs a similar role as the one postulated by Dean and colleagues, suggesting an effect on the muscles’ mechanics in addition to the effects on tendon stress that we have shown currently.

A recent experimental study involving human cadavers supports this line of thoughts as well. Lersch, *et al*.^36^ found that calcaneal inversion and eversion influenced strain profiles within the Achilles tendon more than changes in muscle force distributions. An interesting finding from their study was that eversion of the calcaneus, which reduces Achilles tendon fascicle twist, led to increased heterogeneity of strain distributions within the tendon, especially on the medial side, while the opposite occurs during calcaneal inversion. Results from the present study support this finding. When no fascicle twist was present in our models, corresponding to the eversion case in Lersch study, the medial side of the tendon was loaded disproportionately, while increases in fascicle twist, which corresponded to the inversion case, redistributed this stress and led to more homogeneity of the strain distribution. This demonstrates the role that fascicle twist plays in balancing and redistributing strains within the Achilles tendon.

Non-uniform strain distribution within the tendon has been reported by many in the past. Magnusson, *et al*.^37^ performed *in vivo* experiments by transversely inserting syringe needles into the Achilles tendon. After maximal plantarflexion, needle distortion indicated heterogeneous tendon deformation. Bojsen-Møller and colleagues found shear displacement between the soleus and the medial gastrocnemius during isometric contractions at different knee joint angles^34^. More recently, others^38^ have used ultrasonography based speckle tracking to observe greater displacement in the anterior free tendon compared to the posterior free tendon during passive and active dorsiflexion and while walking. For this phenomenon, computational modelling was used to attribute this behaviour to a combination of differential muscle forces and inter sub-tendon motion^16^, which showed nonuniform displacements between sub-tendons within the Achilles tendon. However, the effects of such nonuniform displacements within sub-tendons on tissue internal stress distribution is still not known. In this study, we have built a separate model that is capable of simulating sub-tendon sliding while measuring tissue stress distribution. The results showed that while twist redistributes stress within the tissue, sliding is related to transmitting force between sub-tendons, hence increasing tissue strength. This result provides a mechanical explanation on the interaction between tendon behaviour and tissue internal structure.

The results of our sensitivity analysis provide context to the role of fascicle twist in tissue strength as compared to the geometrical and mechanical factors of cross-sectional area and stiffness. Increases in cross-sectional areas reduce the average stress across the tendon, providing a better safety margin. Previous work has shown long distance runners have significantly increased cross-sectional areas of the Achilles tendon compared to those in different sports^39,40^. Increases in tendon stiffness result in smaller strains at any given force, reducing the likelihood of tendon strain injuries^41^. Our sensitivity analysis revealed that variations of these three factors by one standard deviation each (±σ), cross-sectional area led to the biggest change in tendon strength (mean change 35%, p < 0.001), followed by stiffness (mean change 28%, p < 0.01). Changes in fascicle twist also led to statistically significant changes in tendon strength (mean change 13%, p < 0.05) but when compared with other factors, this change was the smallest (Fig. 6). Previous studies reported that tendon is a metabolically active tissue that responds to mechanical stimulus and remodels itself by changing cross-sectional area and stiffness^15^. It still remains to be seen if fascicle twist would also change after active training.

There are a number of limitations in this study that bear consideration. First, we modelled the tendon as a hyperelastic material and did not consider time and history dependent viscoelastic changes in tissue material properties. However, the focus of our study was not on time dependent tissue remodelling, but rather tissue strength. In such an instance, the non-linear elastic aspect dominates the overall tissue behaviour^42,43^, hence it is common practice to use hyperelastic material properties. Second, the twist angle implemented was from an idealized fascicle twist, which was generated by twisting the fascicles according to the descriptions from anatomical studies^9,10^. However, a more accurate approach would be to use *in vivo* imaging data, especially considering the possibility of inter-subject variability^6^. Unfortunately, to our knowledge, high fidelity *in vivo* imaging of fascicle twist has not yet been reported. In this study we implemented a number of different twist angles in our model to account for these variations. However, the limitations in the hyperelastic constitutive model and continuum approximation of twist angles mean that the results presented in this study are still qualitative in nature. Nevertheless, development of methods for MRI imaging of tendon fascicle twist is underway. Thus, it is promising that future imaging approaches will provide more accurate ways of describing subject-specific fascicle twists for computational modelling work. Finally, variability between subjects and between twist angles exists in optimized material parameters. A recent anatomical study of the Achilles tendon also found huge variations in twist angles and other tissue characteristics^6^. It is possible that our results reflect such anatomical variations. In fact, we have performed a separate analysis in Appendix I which showed that subject-specific geometry is the main cause of variability. However, the experimental data that we used for material optimization was uniaxial cyclic stretching experiments using cadavers, which has limitations in delineating tissue behaviors completely. Therefore, caution is required in interpreting the material property optimization results.

In conclusion, we have quantitatively analysed the role of fascicle twist in the Achilles tendon using subject-specific FE modelling. Our results indicate that fascicle twist allows strain concentrations developed in the tendon to be redistributed to wider areas, leading to relief of stress concentration and greater effective tissue strength. The modelling framework introduced here can be implemented as subject-specific geometry, optimized material properties, and a range of physiologically reasonable twist angles. Future studies incorporating these approaches may help to design biomechanically informed training and rehabilitation protocols for management of tendon injuries and degeneration^44^.

## Electronic supplementary material

Supplementary Information
