# Supplementary material for: Combining in silico and in vitro experiments to characterize the role of fascicle twist in the Achilles tendon
Source: Sci Rep. 2018 Sep 14;8:13856. doi: 10.1038/s41598-018-31587-z (PMC6138712; doi:10.1038/s41598-018-31587-z)
Supplement: Supplementary file 1 — Supplementary Information [file 41598_2018_31587_MOESM1_ESM.pdf]

Combining *in silico* and *in vitro* experiments to characterize the role of fascicle twist in the Achilles tendon

Vickie B. Shim[1,2*], Geoff G. Handsfield[1], Justin W. Fernandez[1,6], David G. Lloyd[2,3,4,5] & Thor F. Besier[1,6]

1. Auckland Bioengineering Institute, University of Auckland, Auckland, New Zealand
2. Innovations in Health Technology, Menzies Health Institute Queensland, Griffith University, Gold Coast Campus, Australia
3. School of Allied Health Sciences, Griffith University, Gold Coast Campus, Australia
4. Gold Coast Orthopaedics research and Education Alliance
5. School of Human Sciences, University of Western Australia, Australia
6. Department of Engineering Science, University of Auckland, New Zealand

Appendix I

We have performed a linear regression analysis to quantify the influence of subject specific geometry, fascicle twist angles and material parameters on tendon internal stress values using the method from our recent publication[1]. The subject-specific models in our study were generated by morphing an identical generic mesh so they have the same number of elements and nodes. We extracted stress values at numerical integration points (called Gauss points), which occupy the same material locations in each mesh. Then we compared the stress values at those Gauss points for different combinations by varying material properties, geometries and twist angles to see which parameter causes greatest variations in internal stress patterns.

Specifically, we tried the following two combinations– 1) average material properties vs variations of material properties (by varying each parameter by their standard deviation value); 2) average geometry vs. subject-specific geometry (with average material property). Linear regression analysis was performed to determine how the slopes of the regression between stresses at Gauss points (von Mises stress) were influenced by each of above combinations.
We also computed sensitivity values for each variation. Sensitivity was defined as the following according to the previous study by Anderson et al.[2].

$$S = \frac{\%\ change\ in\ slope}{\%\ change\ in\ input\ parameters}$$

The following plot shows that when material parameters were changed by their standard deviation, the slope did not change much, regardless of which parameter was varied (Figure A1, Left). On the other hand, the slope changed dramatically when the geometry was changed from average to subject-specific, indicating that different geometry will lead to different set of parameter values (Figure A1, Right). Moreover, when quantitatively compared using sensitivity values, the stress values were 10 times more sensitive to the geometry than material property values. This clearly shows that the source of variability in our material property optimization was subject-specific geometry and large variations in parameter values between subjects are necessary to capture the anatomical variations in subjects.

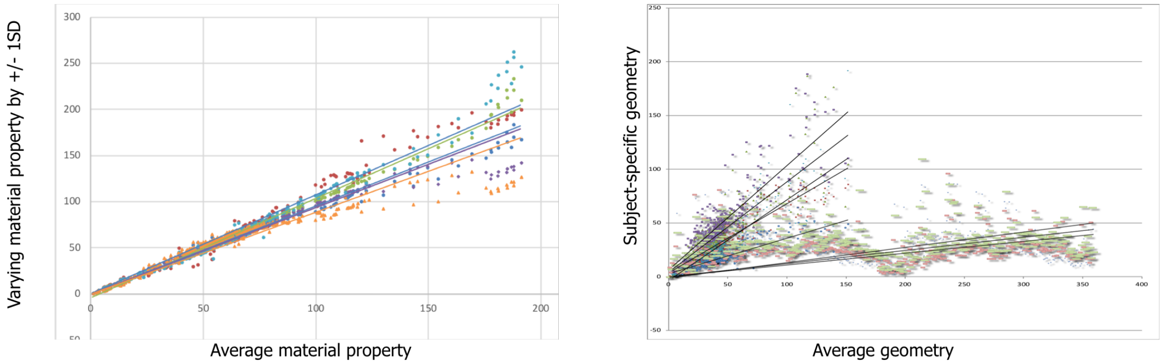

Figure A1. Scatter plots and linear regression line for various combinations for material properties and subject-specific geometries. The left shows the variation in material properties by +/- 1 standard deviation does not change stress values at Gauss points. The right shows that variations in geometry changes stress values at Gauss points dramatically.

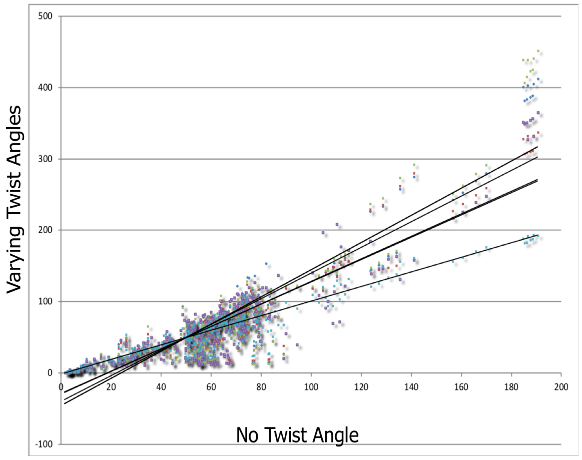

Figure A2. Scatter plots and linear regression lines for variation in twist angles against no twist angle case. Stress values at Gauss point changed as twist angle changed

Since each twist angle leads to different degrees of deformation and subsequent differences in stress pattern, this is reflected in the variations in optimized parameter values between twist angles (Figure A2). As such, we have also performed a linear regression analysis and checked the influence of varying twist angle, specifically we compared stress values at Gauss points at no twist angle with different twist angles. As the plot shown below, the stress values are sensitive to the changes in twist angle, which resulted in different optimized parameter values at different twist angles.

Reference:

1 Hansen, W. *et al*. Achilles tendon stress is more sensitive to subject-specific geometry than subject-specific material properties: A finite element analysis. *Journal of Biomechanics* **56**, 26-31, doi:https://doi.org/10.1016/j.jbiomech.2017.02.031 (2017).

2 Anderson, A. E., Ellis, B. J. & Weiss, J. A. Verification, validation and sensitivity studies in computational biomechanics. *Comput Methods Biomech Biomed Engin* **10**, 171-184, doi:10.1080/10255840601160484 (2007).
